# Oil price pass-through into inflation in Spain at national and regional level

**DOI:** 10.1007/s13209-020-00222-4

**Published:** 2020-10-20

**Authors:** Ligia Topan, César Castro, Miguel Jerez, Andrés Barge-Gil

**Affiliations:** 1 Department of Economics, Universidad Pública de Navarra, Pamplona, Spain; 2 Department of Economic Analysis and Quantitative Economics, Universidad Complutense de Madrid, Madrid, Spain

**Keywords:** Inflation, Deflation, Oil price, Forecasting, Simulation, E31, E37, Q43

## Abstract

Oil price showed sharp fluctuations in recent years which revived the interest in its effect on inflation. In this paper, we discuss the relationship between oil price and inflation in Spain, at national and regional levels, and making the distinction between energy and non-energy inflation. To this end, we fit econometric models to measure the effect of oil price shocks on inflation and to predict them under different scenarios. Our results show that almost half of the volatility of changes in total inflation is explained by changes in oil price. As could be expected, the energy component of inflation drives this effect. We also find that, under the most likely scenarios, 1-year ahead total inflation will be moderate, with relevant differences across regions.

## Introduction

The relevance of oil cost to determine consumer prices is conventional wisdom since the oil shocks of the 1970s, when inflation reached two digits in most industrialized countries and around 25% in Spain. This view has been challenged by several works showing that the influence of oil price on inflation decreased in the last decades (see, e.g., Hooker 2002; De Gregorio et al. 2007). However, in the last years there have been several sharp downward fluctuations in oil prices, with decreases in its 12-month rate around ${-}$ 50% and ${-}$ 30% in January 2015 and January 2016, followed by a sharp 80% increase in January 2017. Our interest in studying the relationship between changes in oil prices stems from this new scenario that has been even exacerbated due to the COVID-19 pandemic.

According to the Spanish Institute of Statistics (hereafter, INE), the 12-month Consumer Price Index (CPI) inflation in Spain stood at negative values during most 2016, with a minimum of ${-}$ 1.1% in April, and recovered during 2017, with a peak of 3% in January and February. There was also a remarkable heterogeneity in inflation among the different regions,^1^ with values ranging from ${-}$ 1.5% in Castile-La Mancha to ${-}$ 0.7% in the Basque Country. The main goal of this work consists in measuring the ‘pass-through’ of changes in oil prices into inflation. Accordingly, we measure and analyze this effect in Spain, at both national and region levels, also making the distinction between the energy and non-energy components of the CPI. Our basic approach is similar to that in Castro et al. (2016): we first fit a times series model relating CPI with oil price. Its dynamic structure implies that the price of crude oil today affects prices in the same and the following month, with no feedback in the opposite direction of Granger (1969) causality (G-causality). The models fitted accordingly are then applied to: (i) decomposing total, energy and non-energy CPIs into two unobserved components, one related to oil prices and the other independent from them; (ii) using these results to compute a variance decomposition; and (iii) computing 12-month ahead inflation forecasts, under different scenarios for oil prices.

On the other hand, we introduce some methodological novelties in comparison with Castro et al. (2016) and other previous works. First, we use a conditional forecasting method which is equivalent to the one used by Castro et al. (2016), but easier to apply with standard software. Second, we provide a variance decomposition method to measure the ‘pass-through’ effect in terms of volatility. Third, we provide a drill-down analysis up to the regional level, which reveals important differences in the pass-through effect across regions.

Some related studies on this topic focus on the relationship between changes in oil price and inflation in Spain, without analyzing differences across regions. For example, Cuñado and de Gracia (2003) focus on the effect of inflation on production using a trivariate VAR analysis, concluding that oil prices Granger-cause economic activity, even when inflation is included in the model.

Álvarez et al. (2011) analyze the impact of oil price shocks in Spain and the Euro Area. They use a Dynamic Stochastic General Equilibrium (DGSE) model and conclude that oil price changes account for more than 50% of the variance of Spanish inflation (45% in the euro area). Castro et al. (2017) analyze this relationship for different industries and four European countries, including Spain. They conclude that Spain is the country with the largest number of industries affected by oil price changes.

To the best of our knowledge, only two studies adopt a regional approach. First, De Dios Tena et al. (2010) focus on improving forecasts of Spanish inflation by aggregating local forecasts, not including oil price variations in their models. Second, Gómez-Loscos et al. (2011) is closer to our analysis, as their goal was to estimate the effect of oil price shocks on the inflation of different Spanish regions with a long-term perspective. They conclude that the effect was stronger in the 70s and increased again at the beginning of the twenty first century, after a spell of low sensitivity.

The structure of this paper is as follows: Sect. 2 describes the dataset and the econometric methods employed. Section 3 presents and discusses the main results for Spain, Sect. 4 does the same for its autonomous regions and cities, and finally, Sect. 5 provides some concluding remarks.

**Table 1:** The data

Notation	Variable	Source
$P_t$	100 times the natural logarithm of total CPI	Spanish Institute of Statistics (INE) (http://www.ine.es)
$E_{t}$	100 times the natural logarithm of energy CPI	INE
$\hbox {NE}_{t}$	100 times the natural logarithm of non-energy CPI	INE
$O_t$	100 times the natural logarithm of Brent price in €/barrel	US Energy Information Administration (EIA) and OECD

## Data and methods

### Description of the sample and data transformations

The dataset used includes monthly series of total, energy and non-energy CPIs in Spain and its 19 administrative regions (*Comunidades Autónomas*) as well as the corresponding monthly Brent spot prices. Since Brent price is quoted in US dollars (USD) and the shopping basket considered to compute the Spanish CPIs is measured in euros, we converted the former to euros.^2^

As Table 1 shows, we denote by $P_t$, $E_t$, $\hbox {NE}_t$ and $O_t$ the total CPI, energy CPI, non-energy CPI and Brent price measured in natural logarithms multiplied by 100, so that their changes can be interpreted as (log) percent rates. Hereafter, we will refer to these variables by their name, dropping the reference to the $\log \times 100$ transformation to avoid cumbersome wording.

**Fig. 1:**
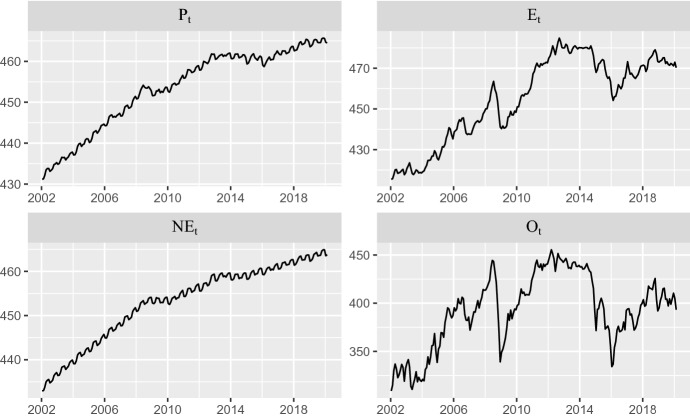
Total ($P_{t}$), energy ($E_{t}$) and non-energy ($\hbox {NE}_{t}$) CPIs in Spain and Brent price ($O_{t}$), Sample: 2002.01–2020.02

Figure 1 displays the profile of the variables described in Table 1. Note that all of them are non-stationary, therefore requiring at least one difference to achieve a stable mean. Total and non-energy CPIs also display seasonal fluctuations, so they need an additional seasonal difference.

Table 2 summarizes the main descriptive statistics of the stationary transformed series, as well as the corresponding *p* values for the ADF (Dickey and Fuller 1981) and KPSS tests (Kwiatkowski et al. 1992). Note that ADF rejects a unit root while KPSS does not reject stationarity, therefore confirming the adequacy of the transformation chosen.^3^

**Fig. 2:**
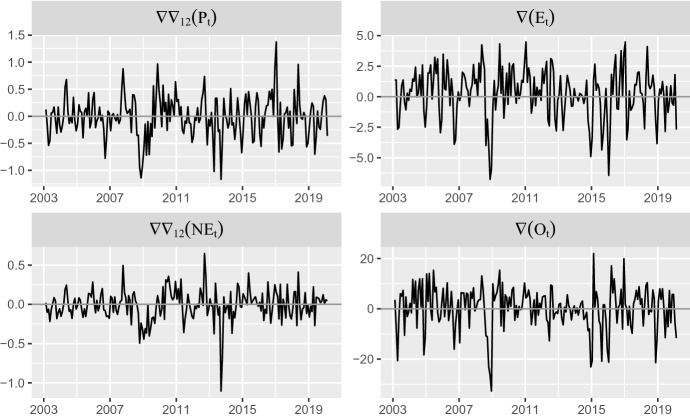
Stationary transformations of the series in Fig. 1, Sample: 2003.02–2020.02

Figure 2 shows the profile of the stationary transformed series. Note that $\nabla \nabla _{12} (P_{t})$ and $\nabla \nabla _{12} (\hbox {NE}_{t})$ can be interpreted as the monthly acceleration of the annual inflation (in log-percent rate). On the other hand, $\nabla (E_{t})$ and $\nabla (O_{t})$ are the monthly log-percent variation rates of Energy CPI and Brent price.

**Table 2:** Descriptive statistics for the stationary series of total $(P_{t})$, energy $(E_{t})$ and non-energy $(\hbox {NE}_{t})$ CPIs in Spain and Brent price $(O_t)$, Sample: 2003.02–2020.02

	$\nabla \nabla _{12} (P_{t})$	$\nabla (E_{t})$	$\nabla \nabla _{12} (\hbox {NE}_{t})$	$\nabla (O_{t})$
Mean	$-$ 0.01	0.24	$-$ 0.01	0.27
SD	0.38	2.03	0.19	8.46
Minimum	$-$ 1.17	$-$ 6.78	$-$ 1.1	$-$ 32.7
Maximum	1.37	4.49	0.65	21.96
*p*-value ADF	0.01	0.01	0.01	0.01
*p*-value KPSS	0.1	0.1	0.1	0.1

$\nabla$ indicates the difference operator, such that $\nabla {y_t}=y_t-y_{t-1}$ and $\nabla _{12}$ indicates the seasonal operator, such that $\nabla _{12}{y_t}=y_t-y_{t-12}$ for any variable $y_t$

### Causality analysis

Most works about oil price pass-through into inflation consider bidirectional causality between both variables and, therefore, use the popular vector autoregressive (VAR) analytic framework. This feedback may be reasonable when the inflation rate corresponds to a large and oil-producing economy such as, e.g., the USA. However, it is hard to believe that inflation in Spain may affect global oil prices.

**Fig. 3:**
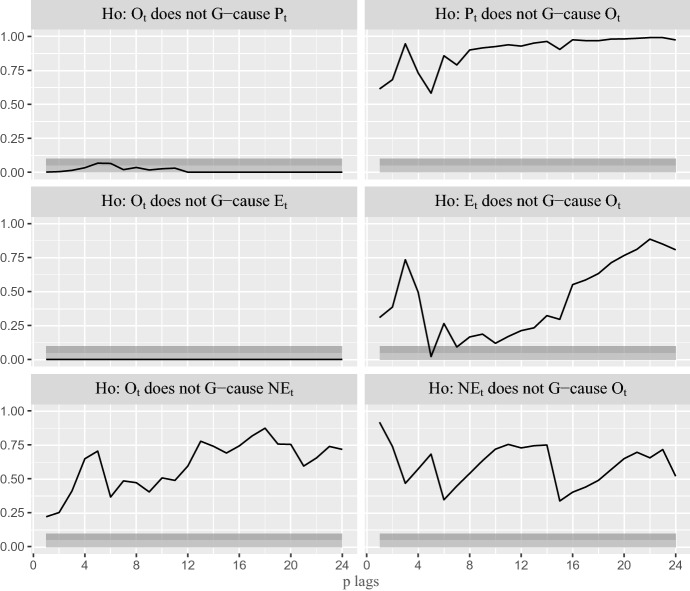
*p*-values for the bivariate G-causality tests between the stationary transformations of CPIs and Brent price. Light shaded areas imply the rejection of the null at the 5% critical level, while dark shaded areas imply rejection at the 10% level, Sample: 2003.02–2020.02

To confirm empirically this idea, a first step in our analysis consists in testing whether the dynamic relationship between changes in Spanish CPIs and changes in oil price is one-way or bidirectional. To this end, Fig. 3 shows the *p* values for the (Granger 1969) test computed for different lag lengths. As expected, the first two rows in this figure indicate strong evidence of G-causality running from oil price to both total ($P_t$) and energy ($E_t$) CPIs, up to 24 lags. On the other hand, the null that the reverse effect is zero is not rejected but for one lag. The last row otherwise rejects the G-causality from oil price to $\hbox {NE}_t$.

These results, which are further confirmed by the cross-correlation analysis in Sect. 3, imply that a VAR specification would not be the best choice to model the relationship between these variables. In particular, a single-output transfer function (TF) model (Box et al. 2015) is better suited because: (i) it is a flexible and efficient representation for a single-direction causal relationship; (ii) allows for instantaneous effects; and (iii) can represent the seasonality of the endogenous variable by means of the ARIMA model for the error term.

## Empirical results for Spain

### Univariate models and transfer function specification

Following Box et al. (2015), we first performed an univariate (ARIMA) identification analysis, which suggested an ARIMA$(1,1,0)\times (0,1,1)_{12}$ specification for $P_t$ and $\hbox {NE}_t$, an ARIMA(2, 1, 0) for $E_t$ and an ARIMA(1, 1, 0) for $O_t$. The corresponding estimation results are shown in Table 3.

**Table 3:** Modeling results corresponding to the ARIMA$(2,1,0)\times (0,1,1)_{12}$ process $(1-\phi _1 L-{\phi _2}L^2) \nabla ^d \nabla ^D_{12}y_t = (1 - \varTheta _1 L^{12}) a_t$, where $y_t$ stands for each one of the series considered, Sample: 2002.01–2020.02

Coefficient	$P_t$	$E_t$	$\hbox {NE}_t$	$O_t$
*d*	1	1	1	1
*D*	1		1	
$\hat{\phi }_1$	0.388	0.469	0.31	0.22
	(0.064)	(0.067)	(0.066)	(0.066)
$\hat{\phi }_2$		0.152		
		(0.068)		
$\hat{\varTheta }_1$	0.824		0.643	
	(0.054)		(0.051)	
$\hat{\sigma }_a$	0.289	1.827	0.186	8.268
$\log -lik$	$-$ 26.908	$-$ 437.733	95.712	$-$ 765.819
AIC	59.816	881.466	$-$ 185.424	1535.638
*Q*(39)(*p* value)	14.489(1)	18.103(0.997)	22.135(0.981)	38.757(0.435)

*d* denotes the regular differences and *D* the seasonal differences. The figures in parentheses are the standard errors unless otherwise indicated. The *Q*(39) statistic is the Ljung and Box (1978) portmanteau test for the null of no residual autocorrelation, computed with the first 39 residual autocorrelations

Note that the residual standard deviation in the oil price model is many times larger than those in the CPI models. This clearly illustrates the fact that oil price is extremely volatile and, in practice, means that its forecasts will be affected by a large uncertainty. The analytic approach suggested in Sect. 3.5 addresses this issue.

Building on the results from the previous ARIMA analysis, we computed the sample cross-correlation function (CCF) between the residuals of the $P_t$, $E_t$ and $\hbox {NE}_t$ ARIMA models and the $O_t$ series prewhitened with the ARIMA model of the corresponding CPI series.^4^

**Fig. 4:**
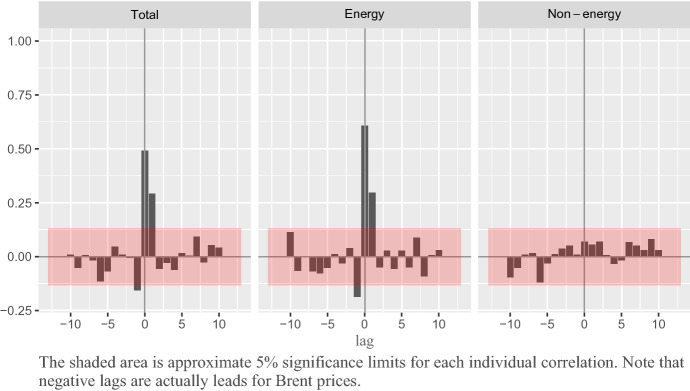
Cross-correlations between the prewhitened series of total ($P_t$), energy ($E_t$) and non-energy ($\hbox {NE}_t$) CPIs and Brent price ($O_t$). Positive lags correspond to the response of $P_t$, $E_t$ and $\hbox {NE}_t$ to shocks in $O_t$, respectively, while negative lags correspond to the inverse causality relationship, Sample: 2002.01–2020.02

The CCFs in Fig. 4 show that:

1. In coherence with the causality analysis in Sect. 2.2, there are no relevant responses of oil prices to shocks in the Spanish CPI (in negative lags).
2. Under the hypothesis that the instantaneous (0-lag) correlation corresponds to the effect of oil price on CPI, Fig. 4 suggests that a unit shock in oil price has a positive and significant instantaneous and 1-lagged effect on total and energy CPI.
3. In the case of non-energy CPI, there is no instantaneous correlation and no significant lagged effect in any direction of causality.

### The response of Spanish inflation to shocks in Brent price

The cross-correlation analysis in the previous section suggests a TF specification relating the current value of CPIs with the current and lagged values of Brent price and an error term with the ARIMA structures already specified for each CPI. The main estimation results are shown in Table 4.

**Table 4:** Estimation results for the TF $CPI_t = ({\omega _0} + {\omega _1}L) O_t + N_t$ with $(1- \phi _1L - \phi _2L^2) \nabla ^d \nabla ^D _{12} N_t = (1 - {\varTheta _1} L^{12}) a_t$, where $CPI_t$ stands for any of the CPIs considered, Sample: 2002.01–2020.02

Coefficient	$P_t$	$E_t$	*NE*
$\hat{\omega }_0$	0.018	0.151	0.001
	(0.002)	(0.009)	(0.001)
$\hat{\omega }_1$	0.013	0.105	0.001
	(0.002)	(0.009)	(0.001)
$\hat{g}$	0.031	0.256	0.003
	(0.002)	(0.011)	(0.002)
*d*	1	1	1
*D*	1		1
$\hat{\phi _1}$	0.187	0.051	0.294
	(0.07)	(0.068)	(0.067)
$\hat{\phi _2}$		$-$ 0.059	
		(0.068)	
$\hat{\varTheta }_1$	0.767		0.642
	(0.05)		(0.052)
$\hat{\sigma }_a$	0.225	1.085	0.186
$\log -lik$	37.469	$-$ 322.143	96.1
AIC	$-$ 64.938	654.285	$-$ 182.2

The figures in parentheses are the standard errors. The sum of the TF weights is the steady-state gain ($\hat{g}$) which measures the total expected change in the model output if we apply a unit impulse to the input

The models for $P_t$ and $E_t$ in Table 4 do not show any symptom of misspecification. In particular, (i) their parameters are highly significant, (ii) the residuals from these TFs do not show relevant correlations, either with the lags of the model input or with their own past, and (iii) their AICs are smaller than those of the ARIMA models in Table 3.^5^ Therefore, they can be considered statistically adequate.

On the other hand, in the model for $\hbox {NE}_t$ the coefficients relating the endogenous variable with the Brent price are small in comparison with their standard errors, the TF gain is not significant at the usual levels and the AIC is larger than the one of the corresponding ARIMA model in Table 3. Therefore, the non-energy component of CPI is indeed insensitive to changes in Brent price.

### Time series decomposition

The transfer functions in Table 4 imply that: (i) changes in total ($P_t$) and energy ($E_t$) CPIs in any month are significantly affected by changes of Brent price ($O_t$) in the same and the previous month, (ii) the effect of $O_t$ on non-energy ($\hbox {NE}_t$) CPI is close to zero, (iii) the response of CPIs to a unit pulse in oil price is transient,^6^ and (iv) the long-term gains of $P_t$, $E_t$ and $\hbox {NE}_t$ are, respectively, $\hat{g}_P=0.031, \hat{g}_E=0.256$ and $\hat{g}_{\mathrm{NE}}=0.003$, being the last one non-significant at the usual levels.

The TF gains measure the sensitivity of CPIs to shocks in oil price. These sensitivities may seem small, but the expected pass-through is given by the product of TF parameters and the corresponding value of $O_t$. Denoting by ${\text {CPI}}_t^O$ the pass-through component for a generic CPI, its estimate would therefore be given by:

1 $$\begin{aligned} \hat{\text {CPI}}_t^O = \hat{\omega _0} \, O_t + \hat{\omega _1} \, O_{t-1} \quad \quad \quad t=2,\ldots ,n \end{aligned}$$

so, if the size of the shocks in $O_t$ is large enough, the pass-through may be substantial.

Building on Expression (1), the part of CPI due to other (non-oil) factors would be given by:

2 $$\begin{aligned} \hat{\text {CPI}}_t^F=\text {CPI}_t - \hat{\text {CPI}}_t^O. \end{aligned}$$

**Fig. 5:**
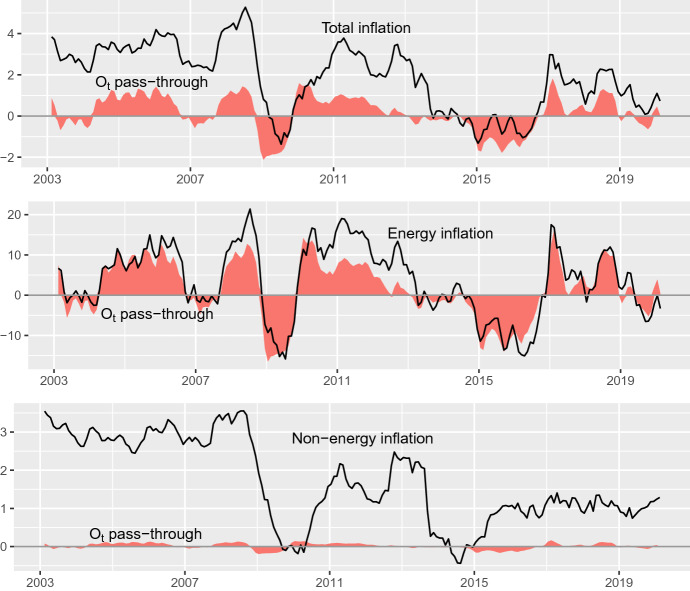
12-Month percentage change of total, energy and non-energy CPI versus the estimated oil price pass-through. These series are standard (non-log) variation rates, Sample: 2003.02–2020.02

Figure 5 displays the profile of total, energy and non-energy inflation versus the pass-through component, computed according to (1). Note that: (i) total and energy inflation display a high degree of comovement (their sample correlations with the pass-through component are 0.710 and 0.843, respectively); (ii) there is virtually no pass-through to non-energy inflation; (iii) the contribution of oil prices to total inflation ranges from 1.811 to ${-}$ 2.119 percentage points in some months, while the contribution to energy inflation ranges from ${-}$ 16.494 to 16.292 percentage points; and (iv) the oil price pass-through is a major determinant of the deflation spells observed in 2009 and 2014–2016. As noted before, the effect of oil prices on inflation is transient, so the duration of these spells is due to relatively long streaks of negative shocks in oil prices. After the end of these streaks, inflation rates recovered positive values in absence of other deflationary factors.

### Variance decomposition

In a dynamic model, variance decomposition is difficult to compute for two reasons: first, because potential non-stationarity of the variables involved and second, because one needs to take into account lagged effects of the exogenous variables. However, the value of $\hat{\text {CPI}}_t^O$ given by (2) accumulates all these effects into a contemporaneous value and, therefore, can be used to compute a simple variance decomposition for the stationary transformation of the CPIs considered.

Re-ordering the terms in (2), we obtain:

3 $$\begin{aligned} {\text {CPI}}_t = \hat{\text {CPI}}_t^O + \hat{\text {CPI}}_t^F \end{aligned}$$

and, applying a stationary transformation to both sides of (3), this expression yields an expression suitable for a variance decomposition, as the terms in the right-hand side of equation would be independent. However, we found more practical to estimate the following regressions:

4 $$\begin{aligned}&\nabla \nabla _{12} \, P_t = \beta _0 + \beta _1 \nabla \nabla _{12}\left( \hat{P}_t^O\right) + \varepsilon _t^P \end{aligned}$$

5 $$\begin{aligned}&\nabla \left( E_t\right) = \beta _0 + \beta _1 \nabla \left( \hat{E}_t^O\right) + \varepsilon _t^E \end{aligned}$$

6 $$\begin{aligned}&\nabla \nabla _{12}\left( \hbox {NE}_t\right) = \beta _0 + \beta _1 \nabla \nabla _{12}\left( \hat{\hbox {NE}}_t^O\right) + \varepsilon _t^{\mathrm{NE}} \end{aligned}$$

This regression-based approach has two advantages. First, it assures the orthogonality of the terms in the right-hand side of the models (4)–(6), so it provides a ‘cleaner’ decomposition. Second, the decomposition problem is cast in the well-known LS regression framework and, in particular, the *R*-squared statistics for these models provide the percentage of the variance of the (stationary transformed) CPI which is explained by the (stationary transformed) pass-through component.

**Table 5:** Variance decomposition, Sample: 2002.01–2020.02

Coefficient	$\nabla \nabla _{12} (P_{t})$	$\nabla (E_{t})$	$\nabla \nabla _{12} (\hbox {NE}_{t})$
$\hat{\beta }_0$	$-$ 0.011	0.153	$-$ 0.01
	(0.019)	(0.08)	(0.013)
$\hat{\beta }_1$	0.937	0.952	1.304
	(0.068)	(0.047)	(0.523)
$R^2$	0.48	0.657	0.025

The figures in parentheses are standard errors. $R^2$ is the determination coefficient, which measures the percentage of variance of the stationary transformation of CPI explained by the regression

The main LS results for models (4)–(6) are shown in Table 5. They imply that 48%, 65.7% and 2.5% of the variance of the changes in total, energy and non-energy CPI, respectively, were explained by the corresponding changes in Brent price.

### Conditional inflation forecasts

As shown in Sect. 3.2, changes in oil price have significant effects on the CPIs and are, therefore, relevant to forecast short-term inflation. On the other hand, anticipating future oil price movements is very difficult, since this variable is very volatile and mainly determined by difficult-to-predict factors, such as geopolitical risks, global and oil-specific demand, oil supply, shocks from financial markets or unexpected shocks like the COVID-19 pandemic. As highlighted in the introduction, changes in oil prices have been quite abrupt recently.

As oil price fluctuations are hard to predict, we considered three different scenarios for the Brent price in February 2021 (see Table 6): the first one corresponds to the maximum Brent price (the extreme increase observed in March 2012), the second one (stable scenario) assumes that there is no variation in oil prices (the same price than in February 2020), and the third scenario corresponds to the minimum Brent price (the extreme decrease observed in January 2001).

**Table 6:** Scenarios for Brent prices (EUR/Barrel) in 2021.02

	Assumed price	12-Month percentage rate
Maximum price	95.0	86.1
Stable	51.0	0.0
Minimum price	22.0	$-$ 56.9

In this framework, conditional forecasting for Brent prices consists in computing the most likely trajectory connecting the past history of $O_t$ (January 2002 to February 2020) with the terminal values for February 2021, as defined in Table 6, taking into account the dynamics of the time series, as described by the model for $O_t$ in Table 3. Therefore, it reduces to computing model-based interpolations for eleven missing values (2020.03–2021.01) in a time series. Castro et al. (2016) solved this problem using a state-space procedure known as ‘fixed-interval smoothing’ (see Anderson and Moore 1979). This method is precise, fast and efficient, but requires specialized software. Because of this, here we use an alternative approach inspired in Box and Tiao (1975) intervention analysis.^7^ It consists in:

1. Building three artificial variables combining the past history of $O_t$, with eleven null values, and each of the terminal conditions defined in Table 6.
2. Estimating an intervention model for each of these variables including: (i) eleven impulse-type intervention variables,^8^ each one of them corresponding to one of the months which values were set to zero; and (ii) an error model with the ARIMA structure given in Table 3.

The idea consists, therefore, in treating the null values as outliers, so the coefficients of the impulse variables are estimates of the most likely value of oil price at the corresponding point of time. After computing these interpolations, the augmented oil price series between January 2002 and February 2021 is used as input to the transfer functions in Table 4 to compute the corresponding inflation forecasts.

**Fig. 6:**
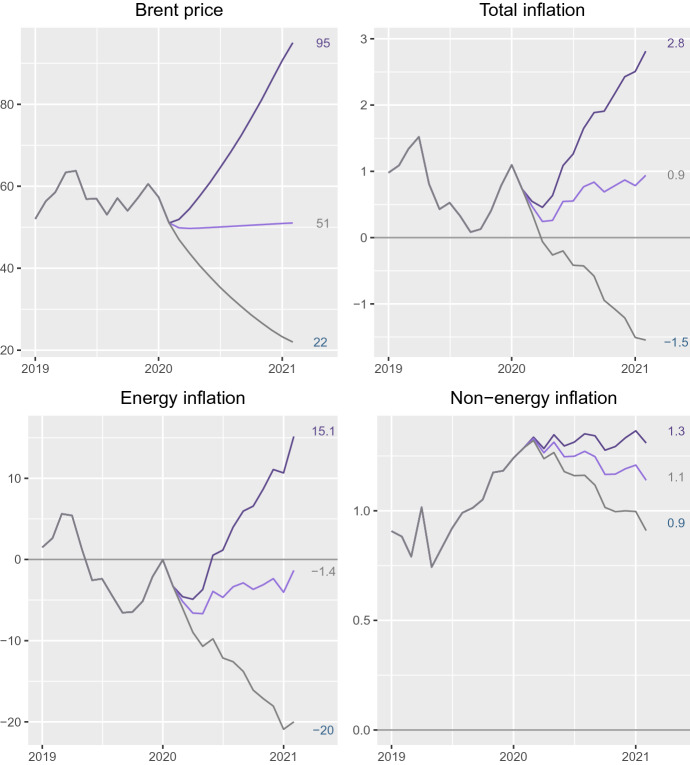
Forecasts between 2020.03 and 2021.02 based on the scenarios depicted in Table 6 for Brent prices and the corresponding 12-month percent rate for total, energy and non-energy CPI in Spain

Figure 6 shows the Spanish inflation forecasts computed in this way. Note that:

1. Under the stable scenario, positive inflation rates are expected although they are well below the 2% inflation which is considered ‘adequate’ by the ECB (the higher rate would be 0.9% in February 2021).
2. Under the extreme increase scenario, inflation projections are again under 2% until the fourth quarter of 2020. A maximum inflation rate of 2.8% would be expected in February 2021.
3. Under the extreme decrease scenario, negative inflation rates will be expected during almost the entire forecasting period (the lower rate would be ${-}$ 1.5% in February 2021).

On the other hand, energy inflation forecasts show that:

1. Under the stable scenario, negative projections of energy inflation are expected (the lower rate would be ${-}$ 6.5% in May 2020).
2. Under the extreme increase scenario, negative energy inflation rates are expected until April 2020. After May 2020, positive rates are expected with a maximum of 15.1% in February 2021.
3. Under the extreme decrease scenario, our results show that energy inflation rates will be always negative with a minimum of ${-}$ 21% in January 2021.

Non-energy inflation forecasts show that:

1. Positive rates are expected in all scenarios.
2. Differences between the different scenarios are very small, in coherence with the small effect of oil prices over this component. The non-energy inflation caused by movements in oil price would vary within a very short interval (0.9–1.25%).

## The response of inflation in Spanish regions to shocks in Brent price

Tables 7, 8 and 9 display some results from the regional transfer function models^9^ for total, energy and non-energy inflation, including (i) the instantaneous and lagged response coefficients; (ii) long-term gains; and (iii) the percentage of variance that can be attributed to changes in oil prices, computed as described in Sect. 3.4. Regions have been sorted in descending order, according to the values of the gain. For comparison purposes, we also included the values for ‘Spain’ as a whole (see Tables 4, 5).

**Fig. 7:**
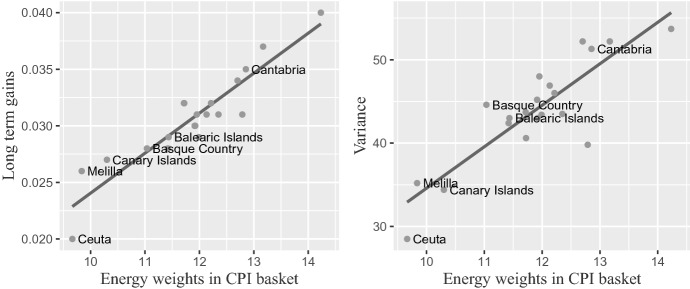
Relationship between Energy weights in CPI basket by Spanish regions (2019) and the long-term gains (left) and the percentage of variance that can be attributed to oil price shocks (right)

The differences in regional sensitivity to oil price shocks in Tables 7, 8 and 9 could be due to three main factors:

1. Road transport costs. Note that the smaller sensitivities correspond to relatively small and isolated geographic regions, such as Melilla, Balearic Islands, Canary Islands and Ceuta, where the effect of road transport costs is probably smaller than that of maritime transportation.
2. Differences in the local CPI shopping basket. Figure 7 displays the relationship between the percentage weight of the total expenditure in energy products within the total coverage of the CPI in 2019^10^ and our estimates of long-term gains and percentage of variance.
3. Energy taxes. Some regions have energy taxes, while others do not. Among the former, there are also important differences in the energy tax rates.^11^

**Table 7:** Long-term effects of Brent inflation on total inflation, Sample: 2002.01–2020.02

	Instantaneous effect	Lagged effect	Long-term gain	Variance
Spain	0.018	0.013	0.031	48.0
*Castile-La Mancha*	*0*.*023*	*0*.*016*	*0*.*040*	*53*.*7*
*Castile-Leon*	*0*.*022*	*0*.*015*	*0*.*037*	*52*.*2*
*Cantabria*	*0*.*020*	*0*.*015*	*0*.*035*	*51*.*3*
Galicia	0.020	0.014	0.034	52.2
Aragon	0.018	0.013	0.032	46.0
Murcia	0.018	0.013	0.032	43.7
Navarre	0.019	0.012	0.032	40.6
Asturias	0.018	0.013	0.031	46.9
Extremadura	0.017	0.013	0.031	43.5
La Rioja	0.019	0.012	0.031	39.8
Andalusia	0.017	0.013	0.030	45.2
Comunitat Valenciana	0.018	0.012	0.030	43.0
Balearic Islands	0.016	0.013	0.029	43.0
Catalonia	0.017	0.012	0.029	43.4
Madrid	0.016	0.012	0.028	42.4
Basque Country	0.016	0.011	0.028	44.6
Canary Islands	0.011	0.016	0.027	34.4
*Melilla*	*0*.*011*	*0*.*016*	*0*.*026*	*35*.*2*
*Ceuta*	*0*.*008*	*0*.*012*	*0*.*020*	*28*.*5*

Summary of estimation and sensitivity results from the regional transfer function models. The regions are sorted according to the long-term gain. The italics area shows the mean of the long-term gain (0.0306) $\pm$ one standard deviation (0.0041) for the Spanish regions

**Table 8:** Long-term effects of Brent inflation on energy inflation, Sample: 2002.01–2020.02

	Instantaneous effect	Lagged effect	Long-term gain	Variance
Spain	0.151	0.105	0.256	65.7
*Castile-La Mancha*	*0*.*169*	*0*.*115*	*0*.*284*	*68*.*3*
*Navarre*	*0*.*168*	*0*.*113*	*0*.*281*	*68*.*1*
*Murcia*	*0*.*169*	*0*.*109*	*0*.*277*	*65*.*6*
*Galicia*	*0*.*165*	*0*.*110*	*0*.*275*	*67*.*1*
*Castile-Leon*	*0*.*161*	*0*.*113*	*0*.*274*	*67*.*3*
Cantabria	0.160	0.105	0.265	65.0
Aragon	0.156	0.107	0.263	65.6
Canarias	0.114	0.149	0.263	58.0
Melilla	0.124	0.136	0.260	55.5
Asturias	0.152	0.106	0.258	66.4
Balearic Islands	0.156	0.100	0.256	61.6
Catalonia	0.147	0.104	0.251	66.5
Comunitat Valenciana	0.150	0.101	0.251	61.2
Madrid	0.149	0.102	0.251	66.3
Andalusia	0.152	0.098	0.249	59.9
*Extremadura*	*0*.*144*	*0*.*094*	*0*.*237*	*57*.*7*
*Basque Country*	*0*.*143*	*0*.*094*	*0*.*237*	*60*.*5*
*La Rioja*	*0*.*138*	*0*.*097*	*0*.*235*	*62*.*3*
*Ceuta*	*0*.*101*	*0*.*128*	*0*.*228*	*50*.*1*

Summary of estimation and sensitivity results from the regional transfer function models. The regions are sorted according to the long-term gain. The italics area shows the mean of the long-term gain (0.2576) $\pm$ one standard deviation (0.0161) for the Spanish regions

**Table 9:** Long-term effects of Brent inflation on non-energy inflation, Sample: 2002.01–2020.02

	Instantaneous effect	Lagged effect	Long-term gain	Variance
Spain	0.001	0.001	0.003	2.5
*Cantabria*	*0*.*002*	*0*.*004*	*0*.*005*	*4*.*0*
*Extremadura*	*0*.*002*	*0*.*003*	*0*.*005*	*2*.*9*
*Basque Country*	*0*.*003*	*0*.*002*	*0*.*005*	*5*.*7*
*Castile-Leon*	*0*.*003*	*0*.*002*	*0*.*004*	*3*.*8*
*La Rioja*	*0*.*003*	*0*.*001*	*0*.*004*	*0*.*6*
*Melilla*	$-$ *0*.*001*	*0*.*004*	*0*.*004*	$-$ *0*.*3*
Andalusia	0.001	0.002	0.003	3.1
Aragon	0.001	0.002	0.003	1.3
Asturias	0.002	0.001	0.003	2.2
Canarias	0.000	0.002	0.003	0.6
Castile-La Mancha	0.002	0.001	0.003	3.0
Comunitat Valenciana	0.002	0.001	0.003	2.1
Galicia	0.002	0.001	0.003	4.1
Murcia	0.001	0.002	0.003	0.9
Navarre	0.002	0.001	0.003	0.5
Ceuta	0.001	0.002	0.003	1.9
*Balearic Islands*	$-$ *0*.*000*	*0*.*002*	*0*.*002*	$-$ *0*.*4*
*Catalonia*	*0*.*002*	*0*.*000*	*0*.*002*	*0*.*1*
*Madrid*	*0*.*001*	*0*.*001*	*0*.*002*	*0*.*7*

Summary of estimation and sensitivity results from the regional transfer function models. The regions are sorted according to the long-term gain. The italics area shows the mean of the long-term gain (0.0033) $\pm$ one standard deviation (0.0012) for the Spanish regions

The tables in “Appendix” report the detailed conditional forecasts for the different regions. Note that:

1. Under the stable scenario, the expected inflation rate at the end of the forecast horizon varies from 1.09% in Catalonia to 0.34% in Ceuta. Therefore, in the most inflationary region expected inflation triple the one in the less inflationary region.
2. Under the extreme increase scenario, expected inflation rate varies between 3.42% in Castile-La Mancha and 1.55% in Ceuta.
3. Under the extreme decrease scenario, all the regions have a negative expected inflation, with the higher deflation being the one in Castile-La Mancha ($-$ 2.23%).
4. Expected inflation depends on both past history and the local sensitivity to oil price. Castile-La Mancha shows the highest gain, so it is the region more affected by the extreme scenarios. In particular, it is the more inflationary (deflationary) region under a ‘extreme increase’ (‘extreme decrease’) scenario.

## Concluding remarks

As noted previously, recent sharp fluctuations in oil prices revived the interest on their effect on inflation. We analyzed this pass-through effect in Spain at both national and regional levels, also making the distinction between energy and non-energy inflation. Our main results show that:

First, CPI changes in any month are affected by changes in oil price in the current and previous month, so a one percent increase in oil price leads to an expected 0.031 percent increase in total inflation, being this effect due to energy inflation (0.256%) rather than to non-energy inflation (0.003%).

Second, the variance decomposition computed in Sect. 3.4 shows that 48%, 65.7% and 2.5% of the variance of changes in total, energy and non-energy CPI, respectively, are explained by the corresponding changes in Brent price.

Third, in the extreme increase scenario Spanish inflation goes over the 2% level at the end of the forecasting period. Under the extreme decrease scenario, negative inflation rates are expected with a minimum of ${-}$ 1.5% at the end of the forecast horizon. Under the same hypotheses, energy inflation would range from 15.1% to $-$ 20.0%, and non-energy inflation would fluctuate between 1.2% and 0.9%.

Fourth, under a stable scenario, positive total and non-energy inflation rates are expected; however, they are under 2%, while energy inflation rates would be negative.

At the regional level, we find important differences in the pass-through effect across regions, being both Castiles and Cantabria the regions where inflation was more responsive to changes in oil price. On the other hand, the islands (Balearic and Canarias) and autonomous cities (Ceuta and Melilla) were the less sensitive ones. Among other factors, this variability can be due to differences in the local shopping basket, geography, climate and energy taxation. Determining properly the relative importance of these factors could be the basis of an interesting topic for further research.

## Appendix

See Tables 10, 11 and 12.

**Table 10:** Conditional forecast analysis for the annual inflation in Spain and its regions for the *extreme increase* scenario for oil price in 2021.02, (95€/barrel)

	March-20	April-20	May-20	June-20	July-20	August-20	September-20	October-20	November-20	December-20	January-21	February-21
*Spain*	*0*.*55*	*0*.*46*	*0*.*63*	*1.09*	*1*.*26*	*1*.*65*	*1*.*89*	*1*.*91*	*2.17*	*2*.*43*	*2*.*51*	*2*.*81*
Castile-La Mancha	0.63	0.54	0.79	1.37	1.58	2.09	2.38	2.23	2.61	2.96	3.03	3.42
Castile-Leon	0.51	0.35	0.54	1.13	1.30	1.85	2.12	2.16	2.53	2.82	2.93	3.30
Galicia	0.47	0.42	0.57	1.12	1.22	1.76	2.11	2.14	2.39	2.64	2.61	3.00
Cantabria	0.54	0.34	0.44	1.02	1.36	1.62	1.96	2.03	2.29	2.46	2.68	2.97
Catalonia	0.65	0.51	0.68	1.03	1.25	1.59	1.88	1.95	2.20	2.44	2.60	2.89
Aragon	0.47	0.31	0.51	0.96	1.14	1.44	1.72	1.87	2.16	2.37	2.47	2.82
Navarre	0.53	0.22	0.43	0.79	0.98	1.38	1.63	1.75	1.96	2.23	2.39	2.81
Murcia	0.66	0.60	0.80	1.19	1.39	1.74	1.93	2.03	2.18	2.51	2.44	2.74
Extremadura	0.55	0.33	0.56	1.05	1.35	1.78	2.04	1.68	1.99	2.42	2.43	2.73
Balearic Islands	0.62	0.51	0.74	1.21	1.44	1.69	1.85	1.88	2.18	2.31	2.49	2.73
Andalusia	0.52	0.50	0.65	1.14	1.31	1.70	1.89	1.86	2.17	2.49	2.46	2.72
La Rioja	0.39	0.17	0.29	0.76	0.91	1.31	1.54	1.64	1.90	2.11	2.23	2.67
Madrid	0.43	0.37	0.55	1.02	1.14	1.53	1.69	1.73	1.92	2.18	2.37	2.65
Comunitat Valenciana	0.38	0.28	0.49	0.99	1.15	1.53	1.75	1.69	1.97	2.20	2.28	2.65
Basque Country	0.59	0.48	0.60	0.98	1.15	1.47	1.70	1.77	1.99	2.24	2.28	2.63
Asturias	0.14	0.14	0.21	0.64	0.80	1.29	1.67	1.70	1.94	2.16	2.13	2.56
Canary Islands	1.13	1.05	1.34	1.57	1.85	2.09	2.14	2.26	2.42	2.44	2.30	2.44
Melilla	0.47	0.33	0.70	0.95	0.87	1.09	1.29	1.35	1.82	1.78	1.94	2.01
Ceuta	$-$ 0.25	$-$ 0.37	$-$ 0.02	0.50	0.56	0.95	1.12	1.02	0.99	1.22	1.38	1.55
*SD*	*0*.*26*	*0*.*27*	*0*.*27*	*0*.*24*	*0*.*29*	*0*.*30*	*0*.*30*	*0*.*30*	*0*.*35*	*0*.*37*	*0*.*35*	*0*.*41*
*Range*	*1*.*38*	*1*.*42*	*1*.*35*	*1*.*07*	*1*.*29*	*1*.*14*	*1*.*27*	*1*.*24*	*1.63*	*1*.*74*	*1*.*66*	*1*.*87*
*IQ range*	*0*.*16*	*0*.*20*	*0*.*22*	*0.18*	*0*.*30*	*0*.*34*	*0*.*32*	*0*.*33*	*0.30*	*0*.*29*	*0*.*26*	*0*.*22*

The regions are sorted according to the forecast in 2021.02

**Table 11:** Conditional forecast analysis for the annual inflation in Spain and its regions for the *stable* scenario for oil price in 2021.02, (51€/barrel)

	March-20	April-20	May-20	June-20	July-20	August-20	September-20	October-20	November-20	December-20	January-21	February-21
*Spain*	*0*.*48*	*0*.*24*	*0*.*26*	*0*.*55*	*0*.*55*	*0*.*77*	*0*.*84*	*0*.*69*	*0*.*78*	*0*.*87*	*0*.*78*	*0*.*94*
Catalonia	0.58	0.30	0.32	0.51	0.57	0.74	0.88	0.78	0.87	0.94	0.95	1.09
Castile-Leon	0.42	0.09	0.09	0.47	0.44	0.78	0.85	0.68	0.84	0.93	0.84	1.02
Castile-La Mancha	0.54	0.26	0.30	0.66	0.65	0.94	1.01	0.65	0.81	0.93	0.79	0.99
Balearic Islands	0.55	0.31	0.39	0.70	0.77	0.87	0.87	0.74	0.88	0.85	0.87	0.97
Basque Country	0.53	0.29	0.26	0.48	0.51	0.67	0.74	0.66	0.72	0.82	0.72	0.92
Madrid	0.37	0.17	0.20	0.52	0.48	0.72	0.72	0.60	0.63	0.73	0.78	0.92
Galicia	0.38	0.18	0.15	0.51	0.42	0.78	0.94	0.78	0.84	0.89	0.69	0.90
Andalusia	0.45	0.29	0.28	0.60	0.61	0.83	0.86	0.66	0.81	0.96	0.76	0.88
Navarre	0.46	0.00	0.04	0.23	0.24	0.47	0.55	0.49	0.53	0.62	0.61	0.87
Aragon	0.40	0.09	0.12	0.39	0.40	0.53	0.63	0.60	0.71	0.74	0.68	0.86
Extremadura	0.48	0.11	0.18	0.51	0.64	0.89	0.98	0.46	0.60	0.85	0.70	0.85
Canary Islands	1.08	0.89	1.03	1.12	1.25	1.34	1.25	1.21	1.23	1.10	0.82	0.83
Cantabria	0.46	0.10	0.02	0.40	0.55	0.62	0.76	0.63	0.70	0.68	0.70	0.82
Comunitat Valenciana	0.31	0.07	0.12	0.46	0.45	0.66	0.71	0.49	0.60	0.67	0.58	0.80
Murcia	0.59	0.37	0.41	0.62	0.65	0.82	0.83	0.75	0.73	0.88	0.64	0.78
La Rioja	0.31	$-$ 0.05	$-$ 0.10	0.20	0.18	0.41	0.47	0.39	0.48	0.52	0.48	0.75
Asturias	0.07	$-$ 0.07	$-$ 0.17	0.10	0.08	0.40	0.61	0.47	0.54	0.59	0.40	0.67
Melilla	0.42	0.16	0.40	0.51	0.28	0.36	0.41	0.33	0.65	0.47	0.48	0.43
Ceuta	$-$ 0.28	$-$ 0.49	$-$ 0.24	0.16	0.11	0.39	0.44	0.24	0.10	0.22	0.27	0.34
*SD*	*0*.*26*	*0*.*26*	*0*.*27*	*0*.*23*	*0.27*	*0*.*24*	*0*.*21*	*0*.*21*	*0*.*22*	*0.21*	*0*.*17*	*0*.*18*
*Range*	*1*.*36*	*1*.*38*	*1*.*27*	*1*.*02*	*1*.*16*	*0*.*98*	*0*.*83*	*0*.*98*	*1*.*13*	*0*.*88*	*0*.*68*	*0*.*75*
*IQ range*	*0*.*16*	*0*.*21*	*0*.*25*	*0*.*16*	*0*.*29*	*0*.*33*	*0*.*25*	*0*.*23*	*0*.*22*	*0*.*27*	*0*.*19*	*0*.*13*

The regions are sorted according to the forecast in 2021.02

**Table 12:** Conditional forecast analysis for the annual inflation in Spain and its regions in the *extreme decrease* scenario for oil price in 2021.02, (22€/barrel)

	March-20	April-20	May-20	June-20	July-20	August-20	September-20	October-20	November-20	December-20	January-21	February-21
*Spain*	*0*.*37*	$-$ *0*.*06*	$-$ *0*.*26*	$-$ *0*.*20*	$-$ *0*.*42*	$-$ *0*.*43*	$-$ *0*.*58*	$-$ *0*.*95*	$-$ *1*.*08*	$-$ *1*.*21*	$-$ *1*.*51*	$-$ *1*.*55*
Ceuta	$-$ 0.33	$-$ 0.66	$-$ 0.56	$-$ 0.30	$-$ 0.50	$-$ 0.37	$-$ 0.47	$-$ 0.82	$-$ 1.10	$-$ 1.13	$-$ 1.22	$-$ 1.28
Catalonia	0.48	0.01	$-$ 0.18	$-$ 0.20	$-$ 0.36	$-$ 0.40	$-$ 0.48	$-$ 0.79	$-$ 0.91	$-$ 1.05	$-$ 1.25	$-$ 1.29
Canary Islands	1.02	0.65	0.60	0.50	0.43	0.32	0.03	$-$ 0.19	$-$ 0.37	$-$ 0.69	$-$ 1.16	$-$ 1.32
Basque Country	0.43	0.01	$-$ 0.22	$-$ 0.20	$-$ 0.38	$-$ 0.41	$-$ 0.55	$-$ 0.83	$-$ 0.97	$-$ 1.08	$-$ 1.37	$-$ 1.34
Balearic Islands	0.46	0.03	$-$ 0.10	0.00	$-$ 0.14	$-$ 0.25	$-$ 0.46	$-$ 0.80	$-$ 0.87	$-$ 1.11	$-$ 1.28	$-$ 1.36
Madrid	0.27	$-$ 0.11	$-$ 0.28	$-$ 0.17	$-$ 0.41	$-$ 0.39	$-$ 0.60	$-$ 0.91	$-$ 1.09	$-$ 1.19	$-$ 1.35	$-$ 1.39
Andalusia	0.35	$-$ 0.01	$-$ 0.23	$-$ 0.13	$-$ 0.34	$-$ 0.35	$-$ 0.54	$-$ 0.95	$-$ 1.02	$-$ 1.09	$-$ 1.49	$-$ 1.56
Extremadura	0.38	$-$ 0.19	$-$ 0.34	$-$ 0.24	$-$ 0.34	$-$ 0.31	$-$ 0.45	$-$ 1.18	$-$ 1.27	$-$ 1.24	$-$ 1.61	$-$ 1.65
Comunitat Valenciana	0.20	$-$ 0.23	$-$ 0.39	$-$ 0.28	$-$ 0.50	$-$ 0.52	$-$ 0.68	$-$ 1.12	$-$ 1.23	$-$ 1.38	$-$ 1.68	$-$ 1.65
Melilla	0.36	$-$ 0.06	$-$ 0.02	$-$ 0.10	$-$ 0.52	$-$ 0.63	$-$ 0.77	$-$ 1.04	$-$ 0.91	$-$ 1.29	$-$ 1.46	$-$ 1.68
Navarre	0.34	$-$ 0.32	$-$ 0.51	$-$ 0.55	$-$ 0.76	$-$ 0.77	$-$ 0.92	$-$ 1.20	$-$ 1.40	$-$ 1.53	$-$ 1.76	$-$ 1.70
Aragon	0.29	$-$ 0.23	$-$ 0.42	$-$ 0.38	$-$ 0.61	$-$ 0.72	$-$ 0.85	$-$ 1.11	$-$ 1.23	$-$ 1.43	$-$ 1.71	$-$ 1.73
La Rioja	0.20	$-$ 0.37	$-$ 0.63	$-$ 0.56	$-$ 0.81	$-$ 0.81	$-$ 0.98	$-$ 1.28	$-$ 1.42	$-$ 1.60	$-$ 1.86	$-$ 1.79
Murcia	0.48	0.06	$-$ 0.14	$-$ 0.16	$-$ 0.36	$-$ 0.43	$-$ 0.65	$-$ 0.96	$-$ 1.22	$-$ 1.30	$-$ 1.76	$-$ 1.81
Asturias	$-$ 0.04	$-$ 0.38	$-$ 0.70	$-$ 0.66	$-$ 0.89	$-$ 0.80	$-$ 0.82	$-$ 1.18	$-$ 1.33	$-$ 1.51	$-$ 1.91	$-$ 1.83
Galicia	0.27	$-$ 0.16	$-$ 0.44	$-$ 0.32	$-$ 0.66	$-$ 0.56	$-$ 0.65	$-$ 1.05	$-$ 1.24	$-$ 1.43	$-$ 1.86	$-$ 1.86
Castile-Leon	0.29	$-$ 0.28	$-$ 0.55	$-$ 0.43	$-$ 0.73	$-$ 0.67	$-$ 0.87	$-$ 1.30	$-$ 1.41	$-$ 1.59	$-$ 1.94	$-$ 1.98
Cantabria	0.34	$-$ 0.25	$-$ 0.58	$-$ 0.45	$-$ 0.56	$-$ 0.75	$-$ 0.86	$-$ 1.24	$-$ 1.42	$-$ 1.70	$-$ 1.92	$-$ 2.02
Castile-La Mancha	0.40	$-$ 0.13	$-$ 0.38	$-$ 0.31	$-$ 0.61	$-$ 0.61	$-$ 0.83	$-$ 1.47	$-$ 1.60	$-$ 1.76	$-$ 2.17	$-$ 2.23
*SD*	*0*.*25*	*0*.*26*	*0*.*29*	*0*.*25*	*0*.*29*	*0*.*27*	*0*.*24*	*0*.*28*	*0*.*28*	*0*.*27*	*0*.*29*	*0*.*27*
*Range*	*1*.*35*	*1*.*32*	*1*.*30*	*1*.*15*	*1*.*32*	*1*.*13*	*1*.*02*	*1*.*27*	*1*.*23*	*1*.*07*	*1*.*01*	*0*.*95*
*IQ range*	*0*.*15*	*0*.*26*	*0*.*33*	*0*.*24*	*0*.*27*	*0*.*31*	*0*.*32*	*0*.*32*	*0*.*37*	*0*.*40*	*0*.*50*	*0*.*45*

The regions are sorted according to the forecast in 2021.02

## Footnotes

1 We consider 17 autonomous regions (Andalusia, Aragon, Balearic Islands, Basque Country, Canary Islands, Cantabria, Castile-La Mancha, Castile-Leon, Catalonia, Comunitat Valenciana, Galicia, Extremadura, La Rioja, Madrid, Murcia, Navarre and Principado de Asturias) as well as two cities with a specific autonomy regime (Ceuta and Melilla).

2 Note that converting oil prices to euro may confound variations in oil prices and exchange rates. Following Castro et al. (2016), we separated both effects by means of a linear approximation and found that the effect of the exchange rate is not statistically significant, so our decision to use oil prices in euro is empirically justified.

3 The null of ADF is that the series has a unit root, while KPSS test assumes that it is stationary. Statistic tests are more conclusive when rejecting the null and, because of this, these tests supplement each other. In this specific case KPSS is determinant, as it rejects stationarity, and ADF provides confirmation by non-rejecting a unit root.

4 As it is known (see Box et al. 2015), this is done because, if one of the prewhitened series is white noise, the cross-correlations are proportional to the corresponding impulse-response coefficients.

5 Besides the *AIC* values shown in Tables 3 and 4, we also considered Schwarz (1978) and Hannan and Quinn (1979) information criteria, which values are nor shown for simplicity.

6 To test for persistent effects, we estimated rational transfer functions, alternative to the models in Table 4, for all the series considered. The estimated denominator polynomial did not have unit roots, which would have implied a persistent effect and, in fact, was non-significant.

7 Another alternative procedure to compute conditional forecasts is that of Pankratz (1989) who provides analytical expressions for ARIMA and VARMA models obtained by a Lagrangean optimization procedure, therefore requiring specialized software.

8 An impulse-type variable has a unit value in the date of the potentially outlying value to be intervened, and zeros in the rest of positions. The corresponding coefficient can therefore be interpreted as the correction required to adjust the outlier to the expected value for the time series. In this case, the values subject to intervention are null, so the coefficients for the corresponding impulse variables are estimates of the expected value for the time series given the past history, the terminal condition and the ARIMA model for the error.

9 The same basic specifications depicted in Table 4 were found statistically adequate for all the regions considered, allowing for some local outliers. In our experience this is not surprising, as the same basic specification is often valid for a set of closely related series.

10 For instance, a typical household in Castile-La Mancha spends 14.2% of its revenues in energy products (mainly fuels for personal transportation, electricity and gas), while in Ceuta this spending is roughly 9.7%.

11 A detailed survey on the complex situation of energy taxes is given by Bergareche (2019).
